# A Primer on Biodefense Data Science for Pandemic Preparedness

**DOI:** 10.1016/j.patter.2020.100018

**Published:** 2020-03-26

**Authors:** Eric Perakslis

**Affiliations:** 1 Department of Biomedical Informatics, Harvard Medical School, Boston, MA, USA

## Abstract

The coronavirus outbreak is sweeping the globe with outbreaks reported on every continent except Antarctica as of March 2020. Data scientists are uniquely and diversely skilled in ways that can be highly effective in minimizing, combatting, and recovering from the impacts of the COVID-19 outbreak. In this Opinion, the basics of biodefense as well as specific opportunities for the data science community to contribute are discussed.

## Main Text

There is no doubt that COVID-19 is an unprecedented challenge for humanity, and one where data scientists can play an active and useful role. In an earlier piece, I provided a brief overview of the field of biodefense with a focus on the importance of risk assessment and resilience, the ability to maintain operations during disasters or other unanticipated crises.^1^ This piece will dig deeper into biodefense policy as well as suggest specific actions that the data science community can take to contribute to COVID-19 resilience, response, and recovery efforts.

### Biodefense Policy in the United States

Starting at the top and looking more deeply into risk and resilience in the United States, much of the policy stems from the Homeland Security Presidential Directive 21, which outlines the policy and strategy for public health and medical preparedness.^2^ Based upon HSPD10, the Biodefense for the 21^st^ Century directive, signed into law by George W. Bush in 2002, the critical components of the plan are biosurveillance, countermeasure distribution, mass casualty care, and community resilience. An excellent source for understanding these initiatives is the Biodefense Policy Landscape Analysis Tool, which provides significant detail on these policies, including the complex web of relationships of roles and responsibilities between and across federal agencies.^3^

One criticism of these policies that is highly relevant to the current outbreak is that they are far too broad and that the original focus, protection from biological weapons, is not aligned with threats such as the current coronavirus outbreak and that an information-driven biosafety strategy that focuses on all threats during peacetime would serve the public better.^4^ To me, these criticisms are highly valid, especially when considering the fractured appearance of the US government responses to COVID-19 to date. What is less clear is whether the government response is fractured due to poor execution or because the plan is indeed a poor fit for peacetime public health threats.

### Resilience

Much of the current peer-reviewed literature on minimizing disruption and ensuring business operations during a crisis and most comprehensive toolkits focus upon ensuring biosafety, which refers to the safe handling of biological agents, or on traditional biodefense, tactics against manmade biothreats.^5^ Traditional approaches to disaster preparedness often focus upon institutional business continuity plans (BCPs). As COVID-19 continues to spread through undocumented community transmission, many have turned to social distancing as their primary prevention and resilience strategy. Globally, organizations are limiting travel, having employees work from home, distributing personal protective equipment, and taking other measures that focus upon containment. These measures are helpful and important, but as we are seeing significant community spread, public health officials are blending their focus from containment only to containment and mitigation, and businesses must look ahead in this manner as well. This outbreak is past the point of prevention, and the response must now focus on minimizing the effects as people get sick.

One of the most effective ways to think about resilience is to recall every cliché you have ever heard about weak links and apply systems thinking. What happens if your child’s school closes and you are forced to quarantine at home with them? Do you have backup, and can you work remotely? What about vendors, contractors, and suppliers that you depend on—is there adequate redundancy in your supply chain? Does your organization already have an experienced virtual meetings culture? Are people used to video conferencing? Are the necessary technology toolsets already in their hands? Have the newest, least experienced, and/or most struggling colleagues been given what they need to do their part?

An addictive quality of humanitarian response actions is the clarity of purpose. There are no endless meetings discussing long-term strategies, and every decision is not accompanied by weeks of handwringing. Priorities are often set on a daily basis. Everyone knows their tasks and where they fit in. Extraneous activity is eliminated as a necessity. Think through priorities with a clear mindset and eliminate the nonessential to free resources for the things that must happen and that need the most help.

### Response

In biodefense strategies, the shift from containment to mitigation initiates the direct-response phase, a shift from prevention to action. Just as the airlines always instruct passengers to place the oxygen mask on themselves before helping others, people must ensure that they themselves and their organizations are secure before attempting to help others. There are many ways that data scientists can be of significant help, and their own communities may be the best place to start. For data scientists who can code, look for opportunities to do that. The same is true for analysts; statisticians; high-content data experts; data policy, governance and regulatory experts; payor and reimbursement policy experts; and all other domains. People looking to help should lead with their strengths but also remember that each of us is more than just the sum of our parts. During the Ebola outbreak of 2014–2015, I was setting up technology at a remote clinic in Sierra Leone. The clinic had not been opened for patients as the water supply had not been inspected or certified. My undergraduate degree happens to be chemical engineering, and I was happy I could help. Be imaginative.

With respect to matching specific expertise and problems, telehealth has great promise and is being touted as a solution, but the current availability is spotty geographically, and rapid implementation will need expert guidance. Specifically, regulatory, geolocation, and reimbursement expertise are needed for telehealth to reach its full potential during this outbreak. For those with expertise in research and epidemiology, you are likely already in high-demand and activated. If not, as the response to coronavirus will be managed primarily within communities, contacting your state, town, county, district board of health, or equivalent may yield instant opportunity to be of assistance. If you work in biomedical product research and development, your company is likely concerned and developing plans to ensure the resilience of clinical trial sites and participants. By definition, patients in clinical trials all have underlying health conditions, and many have health conditions that will put them at high risk of serious effects if infected with the coronavirus. Shortages of drugs and drug manufacture ingredients are already putting the clinical supply chain at risk. Trial deviations and the need to adapt trial designs are all possibilities that will require biostatistical and data science support.

The list goes on and on. Communications strategies must be resilient, and facts must be clear and substantiated. Synthetic and *in silico* modeling of response, outbreaks, supply chain, transmission, and almost every other aspect must be available when and where needed. The tools of digital disease detection are reasonably strong, but if you have ideas that will make them stronger, contact groups such as HealthMap (https://healthmap.org/en/) and share your ideas. Misinformation campaigns must be countered. Campaigns such as “flatten the curve” that are making the rounds on social media are based upon solid facts and can be very useful in educating all of us in the importance of slowing the transmission of the virus regardless of how far it has already gone. Further, anything that can be done to educate patients and caregivers in ways that preserve precious resources such as coronavirus test kits is important. Cyberchondria is a real thing, and many people will be seeking care with minor symptoms and low risk of actual infection.

For coders and epidemiologists, there are already very strong and dedicated platforms for building, modifying, and deploying mobile applications for coronavirus tracking and response use cases. A toolset that I have personally used over the years for rapid development and deployment is CommCare by Dimagi, and they have already built a toolkit and guide specifically for COVID-19 outbreak response.^6^ Another excellent toolset that has been crowdsourced specifically for coronavirus response is the Coronavirus Tech Handbook.^7^ These are not the only toolsets; there are others. Pick the one that fits your immediate needs best and get to work. The important thing is not to waste precious time building things from scratch in outbreak settings when time is of the essence. Reach for something that works and get it into the hands of those that need it. Of course, these are just examples, and the actual opportunities are countless. Use your professional and social networks to find the right opportunity to help. If you are not the best fit, use your network to matchmake between people and problems. Every solution counts.

### Recovery

One fascinating attribute of infectious-disease outbreaks is that it can be challenging to determine when or how they will end. In the 2014–2015 Ebola outbreak in West Africa, the epidemic ended quite abruptly and somewhat unexpectedly, and the response went rapidly from start-up mode to shut-down mode.^8^ My last trip during that outbreak focused upon rebuilding local infrastructure to enable the local health systems to get back to full operation, including the possibility of an Ebola-infected patient presenting and seeking care. Health systems being overrun by infectious-disease outbreaks is not limited to low- and middle-income countries (LMICs). In the US, we have seen seasonal flu outbreaks severe enough that hospitals have had to erect tents to manage the overflow of patients. Unavoidably, healthcare workers will be infected. Clinics will be low on staff and supplies, and some clinics will be forced to close and transfer their burden elsewhere. Re-establishing services and adequate staffing levels and helping institutions through the fragile state of re-opening and expansion is the third domain of necessity and opportunity.

After truly catastrophic events, communications must be re-established and be clear and authoritative. Schools and places of business will need to be ready to be re-opened. Travel and transportation infrastructure must be re-established, and all of these activities must be supported through fragile initial states. This may require new data systems. During the rebuilding period after Ebola, we were tasked with readying clinics for traditional services but also to be prepared for the possibility of an Ebola-infected person to show up seeking care. Specially designed triage applications that walked newly trained health workers through algorithmically guided case definition steps were an essential element of rebuilding the healthcare system.

Another essential element of active response is following through on projects that are likely to be needed during the next outbreak or pandemic. In the modern world, it is amazing how fast priorities change and people move on. Following the rapid shutdown of the Ebola response in West Africa, many loose ends were dropped only to be resurrected 2 years later for the Ebola response in the Democratic Republic of Congo (DRC). Had teams been given the resourcing to finish, package, and archive complete solutions, months could have been saved at the beginning of the DRC outbreak. Just as the Strategic National Stockpile in the US ensures the supply of potentially life-saving drugs and medical supplies, we need a technology and data stockpile equivalent that is properly maintained and ready to go when the next outbreak strikes. Too much time is wasted in the earliest parts of an outbreak when the curve is furthest from being flat.

This is also a good time to simply “show up” physically to lend whatever type of assistance your local clinic or public health service needs. At this point, the risks of infection should be greatly decreased, and many hands make light work. Whether it is coding, data analysis, swinging a hammer or paintbrush, doing a supply run, or helping document and publish data and/or care and research findings, this should be a time of not just recovery but of improvement. It is well known that strong, resilient health systems are and will always be the first line of defense against epidemics. The goal of this reconstruction pause should not be to return them to prior capability but to make them stronger and ready for the next outbreak. It may be here soon enough.

Lastly, first and foremost before considering or acting on anything suggested above, take care of yourself, your family, your livelihood, and your community first. The fewer of us who get sick, the more of us who are available to help others before, during, and after the acute phases of this outbreak and the essential rebuilding phases that follow.
